# The clinical value of iodine-125 seed implantation in the treatment of iodine-refractory differentiated thyroid carcinoma

**DOI:** 10.3389/fendo.2024.1327766

**Published:** 2024-04-15

**Authors:** Qin Wan, Liling Tan, Xinlan Tang, Wenjun Wang, Yu Su, Zhen Wu, Mengmeng Ke, Zhijun Chen

**Affiliations:** ^1^ Department of Nuclear Medicine, Jiangxi Cancer Hospital, Nanchang University, Nanchang, Jiangxi, China; ^2^ Department of Nuclear Medicine, The Second Affiliated Hospital of Nanchang University, Nanchang University, Nanchang, Jiangxi, China

**Keywords:** iodine-refractory differentiated thyroid carcinoma, iodine-125 particles, brachytherapy, iodine radioisotopes, thyroglobulin

## Abstract

**Objective:**

To explore the clinical benefits of ^125^I seed implantation for iodine-refractory differentiated thyroid cancer (RAIR-DTC).

**Methods:**

A retrospective analysis was conducted on 36 patients with RAIR-DTC who underwent radioactive ^125^I seed implantation from January 2015 to February 2022, involving 73 lesions. Prescription dose: 80~120 Gy. All cases were followed up at 1, 3, and 5 months postoperatively to monitor changes in tumor size, serum thyroglobulin (Tg), and serum anti-thyroglobulin antibody levels in thyrotropin-inhibited states, pain scores, and postoperative adverse reactions. The data were processed and analyzed using IBM SPSS 26.0. LER (Local Effective Rate) and LCR (Local Control Rate) were expressed as n (%), tumor diameter, Tg, and pain scores were represented as Median (Q1, Q3). Pairwise comparisons were conducted using the Wilcoxon signed-rank test, and a p-value of less than 0.05 indicated statistical significance.

**Results:**

Tumor size was significantly reduced after treatment (all P < 0.001): tumor length diameters were 32.67 (17.70, 45.72) mm, 27.45 (12.30, 39.98) mm, 20.70 (11.98, 37.58) mm, and 20.39 (10.56, 33.20) mm in the preoperative, 1-, 3-, and 5-months postoperative periods, respectively. Additionally, two consecutive post-treatment results were more minor and statistically significant than the previous results (P < 0.001). The LER at 1-, 3-, and 5-months post-surgery was 23.73%, 38.98%, and 52.54%, respectively, while the LCR at the same time points was 98.31%, 96.61%, and 94.92%, respectively. Patients’ serum Tg levels decreased significantly after surgery. (P < 0.001). Serum Tg levels were measured before surgery and 1-, 3-, and 5-months post-surgery. The results showed that serum Tg levels were 249.45 (79.39, 4718.75) ng/ml, 193.40 (44.53, 2829.00) ng/ml, 192.10 (25.58, 1758.00) ng/ml, and 136.25 (16.57, 1553.25) ng/ml, respectively. Two consecutive post-treatment results were more minor and statistically significant than the previous results (P < 0.001). The patients’ pain symptoms were significantly relieved after ^125^I brachytherapy (P < 0.001). The pain scores before ^125^I seed implantation and at 1, 3, and 5 months after the operation were 5.00 (4.00, 6.00), 3.00 (2.25, 4.00), 2.00 (2.00, 3.00), and 2.00 (1.00, 3.00), respectively.

**Conclusion:**

Most lesions treated with ^125^I seed implantation in RAIR-DTC patients showed shrinkage and improved pain symptoms.

**Clinical trial registration:**

https://www.clinicaltrials.gov, identifier NCT06362772.

## 1 Introduction

Thyroid cancer (TC) is the most common malignant tumor of the endocrine system, and its incidence is increasing (1). Over 90% of cases are differentiated thyroid cancer (DTC). While the majority of DTC cases have a favorable prognosis, with a 10-year survival rate of 90% (2), a subset of patients experience recurrence or distant metastases even after surgery, ^131^I therapy, and thyrotropin (TSH) inhibition therapy (3), and progress to radioactive iodine-refractory differentiated thyroid cancer (RAIR-DTC) (4).

Current therapies for RAIR-DTC, including targeted therapy and external beam radiotherapy, have advantages and disadvantages. Although the targeted drugs sorafenib and lenvatinib are approved for treating RAIR-DTC (5), the impact on overall survival is insignificant (6). There are apparent drug resistance and adverse reactions (5), such as hand-foot syndrome, myelosuppression, hypertension, proteinuria, and diarrhea (7), which make it challenging to meet clinical needs. Molecular targeted drugs, such as mitogen-activated protein kinase (MAPK) inhibitors and phosphatidylinositol 3-kinase (PI3K)/AKT inhibitors, have the potential to redifferentiate RAIR-DTC (8). Although the combination of ^131^I therapy after redifferentiation therapy showed positive effects in RAIR-DTC, long-term follow-up data are lacking. Issues such as population screening, exploration of individualized treatment, and criteria for evaluating therapeutic response remain to be addressed. External beam radiotherapy cannot continuously affect the complete division cycle of the tumor; it damages the surrounding tissue. In addition, TC cells are hypersensitive to external beam radiotherapy (9), limiting local control’s effectiveness.

After Whitmore (10) successfully implanted ^125^I seeds to treat prostate cancer in 1972, ^125^I seed implantation became increasingly popular after the success of TPS in the 1980s. Over the last three decades, due to its effective local control, minimally invasive nature, low complication rates, and good tolerability, ^125^I seed implantation has been widely utilized in treating malignant tumors such as prostate and lung cancer (11). This study aims to explore the application of ^125^I seed implantation in treating bone, lymph node, and lung metastases in RAIR-DTC. Further details are provided below.

## 2 Materials and methods

### 2.1 Clinical data

Data from 36 RAIR-DTC patients hospitalized at Jiangxi Cancer Hospital from January 2015 to February 2022 were retrospectively collected. The average age was 57.39 ± 14.76 years, with 14 males and 22 females. A total of 73 lesions were recorded, including four local recurrences and metastases in various parts of the body: 19 in lymph nodes (8 central, three lateral cervical, two supraclavicular, and six mediastinal), 4 in lung (3 in the left lung and 1 in the right lung), 4 in pleura, and 42 in bones (1 in the mandible, 18 in the vertebrae, 4 in the sternum, 6 in the ribs, 2 in the extremities, 11 in the pelvis). Of the bone metastases, 28 had bone destruction and soft tissue formation, while 14 had bone destruction only. The staging of patients is based on the American Joint Committee on Cancer's 8th edition thyroid cancer staging system (12). The Jiangxi Cancer Hospital Ethics Committee approved all treatment plans. In advance of the surgery, patients were informed about their condition, the expected efficacy of ^125^I seed implantation therapy, alternative treatments such as external beam radiotherapy and chemotherapy, as well as potential side effects and toxic effects. The patient signed an informed consent form after accepting the treatment plan. A total of 1,971 ^125^I seeds were implanted. The median number of particles injected per lesion was 20 (range 4 to 146) with a median radioactivity of 2.59 × 10^7^ Bq (range 1.85 × 10^7^ to 3.33 × 10^7^ Bq). **Table 1** provides detailed baseline data on the patients and foci.

**Table 1:** Patient characteristics(n=36).

Characteristics		No	%
Sex			
Male		14	38.89
Female		22	61.11
Age			
≤60		18	50.00
>60		18	50.00
Histopathology			
PTC		24	66.67
FTC		12	33.33
Cancer Stage			
Age<55Y	I	8	22.22%
	II	6	16.67%
Age≥55Y	I	0	0.00%
	II	2	5.56%
	III	3	8.33%
	IVa	2	5.56%
	IVb	15	41.67%
KPS			
≤80		5	13.89
>80		31	86.11
Previous surgeries			
0		1	2.78
1		16	44.44
2		13	36.11
≥3		6	16.67
Previous radiotherapies			
0		32	88.89
1		1	2.78
2		3	8.33
Previous chemotherapy			
Yes		1	2.78
No		35	97.22
Previous ablation			
Yes		1	2.78
No		35	97.22
Type of lesions			
Local recurrence		4	5.48
Lymph node metastasis		19	26.03
Pulmonary metastasis		4	5.48
Bone metastasis		42	57.53
Pleural metastasis		4	5.48
Type of bone metastasis			
Soft tissue formulation		28	66.67
Bone destruction only		14	33.33

PTC, Papillary Thyroid Carcinoma; FTC, Follicular Thyroid Carcinoma; KPS, Karnofsky Performance Scale; Y, years.

#### 2.1.1 Critical eligibility criteria included

DTC was confirmed by pathology, with local recurrence or distant metastasis of residual thyroid cancer and conforming to any of the following RAIR-DTC criteria: (1) no uptake of ^131^I in the initial ^131^I treatment; (2) loss of iodine uptake capacity in previously functional iodine-avid lesions; (3) disease progression after ^131^I therapy, including gradual enlargement of the lesion and continuously increasing levels of serum thyroglobulin (Tg).

#### 2.1.2 Critical exclusion criteria were as follows

Patients with severe physical diseases, such as severe dysfunction of the cardiac, pulmonary, hepatic, or renal systems, poor compliance, inability to tolerate ^125^I seed implantation, severe acute infectious or chronic infection with acute exacerbation, severe coagulopathy that may lead to serious complications like infection or bleeding, pregnant or lactating women affecting fetal and infant growth and development, patients with cachexia or expected survival of ≤3 months and patients with positive serum anti-thyroglobulin antibodies (TgAb) that may impact the assessment of treatment efficacy.

### 2.2 Materials

Tianjin Said Biopharmaceutical Co., Ltd. supplies ^125^I seeds, specifically seed model 6711-99. Physicists verify the quality of seeds by conducting quality control and sampling at least 10% of the seeds (100% if there are fewer than five seeds). They ensure that the seed radioactivity is within a ±5% deviation to meet the required standards for seed quality. Beijing Atomic Hi-Tech Co., Ltd. provided the seed implantation device, and the 18G seed implantation needle was provided by Hakko International Trading (SHANGHAI) Co., Ltd. The CT scan was performed using a SPECT/CT Symbia T2 from Siemens Healthineers, Germany, with a slice thickness of 5 mm, a slice pitch of 2 mm, a current of 48 mA, and a voltage of 130 kV. The KL-SIRPS-3D model from Beijing Astro Technology Ltd. was used as the treatment planning system.

### 2.3 Method

**Figure 1:**
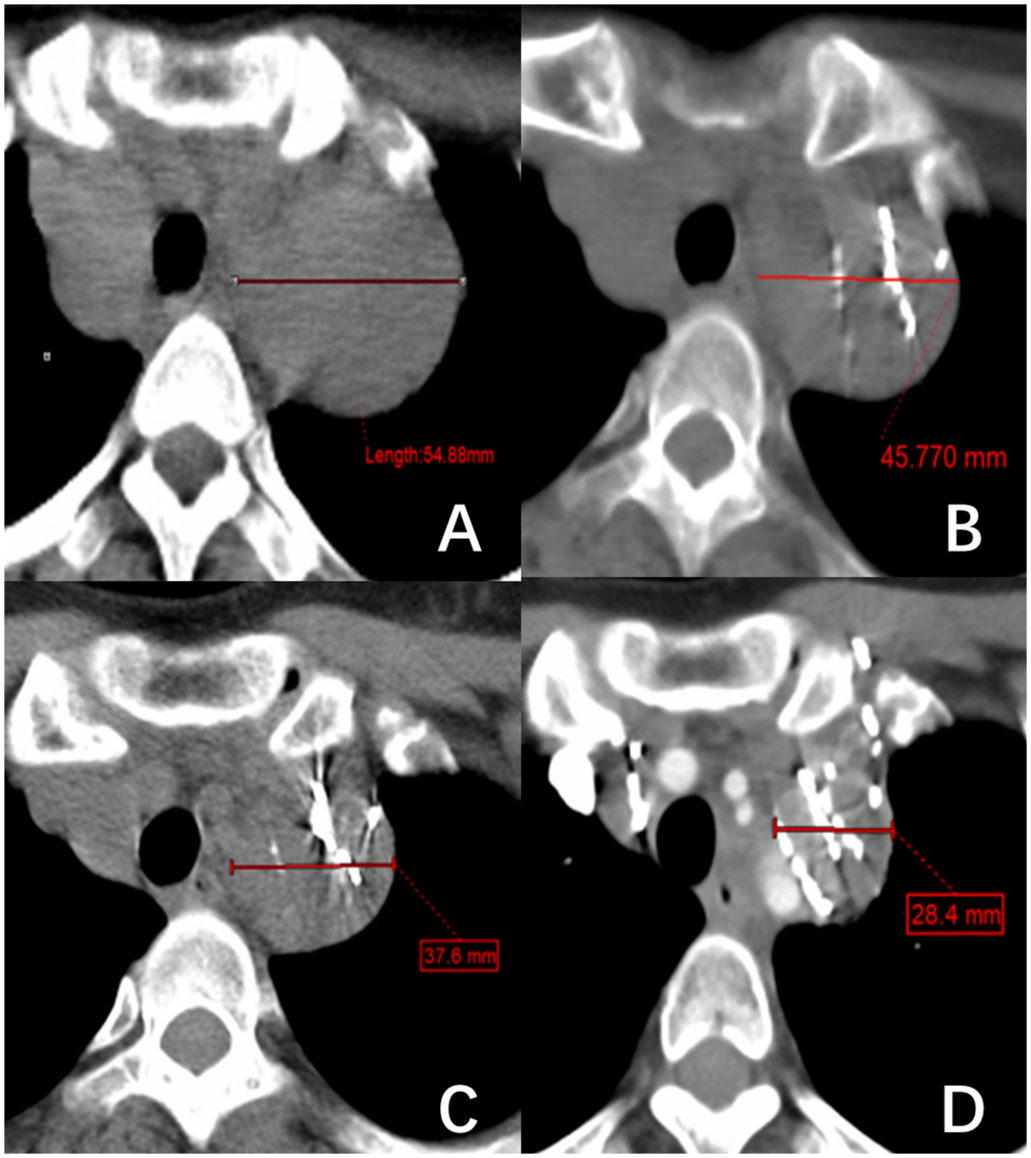
A right superior mediastinal lymph node metastasis of thyroid follicular papillary carcinoma. **(A–D)** show the changes in lymph node short diameter before ^125^I brachytherapy (54.88 mm) and after treatment at one month (45.77 mm), three months (37.6 mm), and five months (28.4 mm), respectively.

**Figure 2:**
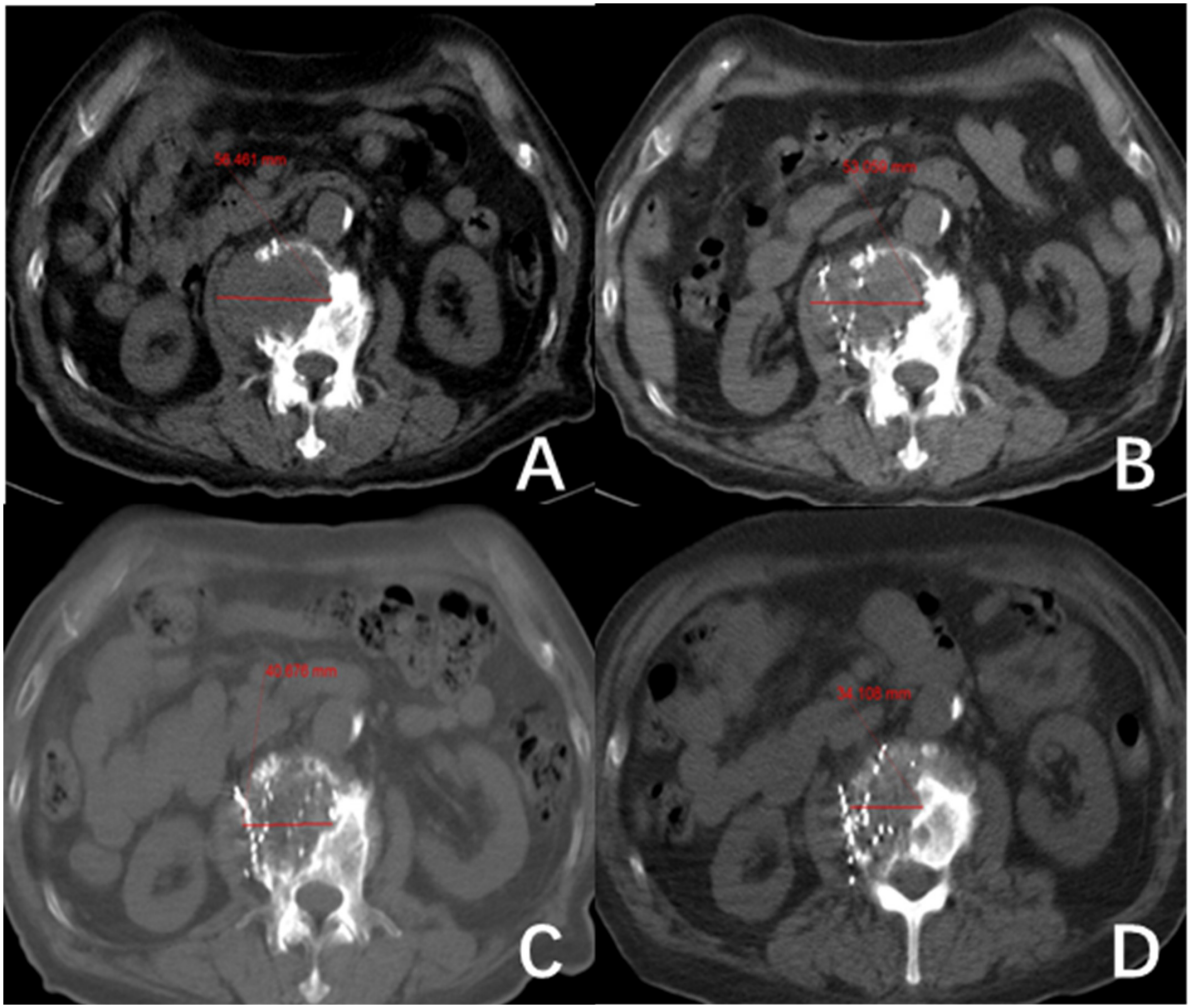
The second lumbar vertebra shows metastasis from follicular thyroid carcinoma (FTC). **(A–D)** show the changes in length and diameter of paraspinal soft tissue metastasis before 125I seed implantation (56.46 mm) and after treatment at one month (53.1 mm), three months (40.68 mm) and five months (34.11 mm), respectively.

All patients received ^125^I brachytherapy treatment and follow-up care at the Department of Nuclear Medicine within Jiangxi Provincial Cancer Hospital. The preoperative evaluation process includes assessments of coagulation function, liver and kidney function, cardiopulmonary function, and local CT scans. A collaborative effort between physicians and physicists was undertaken to establish a treatment regimen with a prescription dose ranging from 80 to 120 Gy. For individuals who have undergone external beam radiotherapy within five months, a dose of 80 Gy is recommended. For individuals with a history of external beam radiotherapy lasting more than five months or those who have received a cumulative dose of ^131^I therapy exceeding 2.22*10^10^ Bq, a dose of 100 Gy is recommended. Patients who did not fall into these specific categories were prescribed 120 Gy. There was a difference in the activity level among ^125^I particles implanted at different locations. The activity level was 1.85 x 10^7^ Bq for near-surface lesions, 3.33 x 10^7 Bq for bone metastases, and 2.22-2.96 x 10^7^ Bq for other metastatic sites. The CT-guided ^125^I seed implantation procedure was conducted following the prescribed treatment regimen. Subsequent evaluations were scheduled at 1-, 3-, and 5-months post-surgery, focusing on lesion dimensions, serum Tg levels, pain levels, and adverse reactions. Lesion size was determined using CT imaging, with a total of 73 RAIR-DTC metastases from various locations included in the study. Measurements were conducted for 59 metastases, excluding 14 lesions that solely impacted bone tissue without associated soft-tissue hyperplasia. This rendered the assessment of treatment efficacy based on tumor size alterations unfeasible. Lymph nodes were assessed based on their shortest diameter (**Figure 1**), while other target lesions were evaluated based on their longest diameter (**Figure 2**). Each measurable lesion underwent an average measurement derived from a minimum of three readings, with all measurements conducted by the same individual to minimize measurement discrepancies.

Response Evaluation Criteria in Solid Tumors (RECIST 1.1) (13) were used to assess treatment response. Local Effective (LE) was defined as a 30% or greater reduction in the longest diameter of the targeted lesion. Progressive Disease (PD) was defined as a 20% or greater increase in the longest diameter of the targeted lesion, while Stable Disease (SD) was defined as being in between. The Local Efficacy Rate (LER) and Local Control Rate (LCR) were calculated by recording the number of lesions classified as LE, SD, and PD at months 1, 3, and 5. (LER = LE/total number of lesions × 100%; LCR = (LE + SD)/total number of lesions × 100%).

Use the Visual Analogue Scale to record patients’ pain scores. The scale ranges from 0 to 10 points, with pain increasing incrementally. 0 indicates no pain, 1-3 indicate mild pain that is still tolerable and does not interfere with sleep or normal life, 4-6 indicate moderate pain that interferes with sleep and requires analgesic medication to alleviate it, and 7-10 show intense and intolerable pain that seriously interferes with sleep and diet and requires strong analgesic drugs.

Postoperative adverse reactions, including infection, bleeding, pneumothorax, bone marrow suppression, and seed displacement, were recorded. Radiation injury was graded according to the Radiation Therapy Oncology Group (RTOG) and European Organization for Research and Treatment of Cancer (EORTC) toxicity criteria (14).

### 2.4 Statistical analysis

The data were analyzed using IBM SPSS 26.0. The count data (n%) and skewed measures [Md (Q1, Q3)] are presented. Group comparisons were conducted using the paired Wilcoxon rank-sum test, with statistical significance set at P < 0.05.

## 3 Results

The diameters of the tumor lengths were 27.45 (12.30, 39.98mm), 20.07 (11.98, 37.58mm) and 20.39 (10.56, 33.20mm) at months 1, 3, and 5 after ^125^I seed implantation, respectively. These values were significantly reduced compared to the initial diameter of 32.67 (17.70, 45.72mm) before treatment (Z= -6.227, -6.272, -6.189; all P < 0.001). Two consecutive post-treatment results were more minor and statistically significant than the previous results (P < 0.001). At one month postoperatively, there were 14 cases of LE, 44 cases of SD, and 1 case of PD. At three months postoperatively, there were 23 cases of LE, 34 cases of SD, and 2 cases of PD. At five months postoperatively, there were 31 cases of LE, 25 cases of SD, and 3 cases of PD. The LER at 1, 3, and 5 months postoperatively was 23.73% (14/59), 38.98% (23/59), and 52.54% (31/59), respectively. The LCR was 98.31% (58/59) at one month, 96.61% (57/59) at three months, and 94.92% (56/59) at five months postoperatively. The interquartile range includes the data [Md(Q1, Q3)]. Please refer to **Table 2** for further details.

**Table 2:** The efficacy of radioactive ^125^I seed implantation.

Follow-up time	LE	SD	PD	LER(%)	LCR(%)
One month	14(23.73)	44(74.58)	1(1.69)	23.73	98.31
Three months	23(38.98)	34(57.63)	2(3.39)	38.98	96.61
Five months	31(52.54)	25(42.37)	3(5.08)	52.54	94.92

The number of lesions is expressed as n(%); LE: Local Effective; SD: Stable Disease; PD: Progressive Disease; LER: Local Effective Rate (LER = LE/total number of lesions × 100%); LCR: Local Control Rate (LCR = (LE + SD)/total number of lesions × 100%).

The serum Tg levels of 36 patients were measured before and after ^125^I seed implantation. The levels were measured in the 1st, 3rd, and 5th postoperative months. Specifically, the serum Tg levels were 249.45 (79.39, 4718.75) ng/ml before treatment, 193.40 (44.53, 2829.00) ng/ml in the 1st month, 192.10 (25.58, 1758.00) ng/ml in the 3rd month, and 136.25 (16.57, 1553.25) ng/ml in the 5th month. The results showed that the serum Tg level decreased significantly after treatment (Z = -3.881, -4.587, -4.823, all P < 0.001). Furthermore, serum Tg levels had returned to normal or were approaching normal in some patients. Two consecutive post-treatment results were more minor and statistically significant than the previous ones (Z = -3.661, -3.931, and P < 0.001). Please refer to **Table 3** for further details.

**Table 3:** The serum Tg level is associated with radioactive ^125^I seed implantation.

Follow-up time	Tg(ng/ml)	Z	P
Preoperative	249.45(79.39,4718.75)	–	–
One month	193.40(44.53,2829.00)	-3.881	<0.001*
Three months	192.10(25.58,1758.00)	-4.587	<0.001*
Five months	136.25(16.57,1553.25)	-4.823	<0.001*

*Compared with preoperative.

The pain scores of 36 patients were recorded at months 1, 3, and 5 after treatment. The scores were 3.00 (2.25, 4.00), 2.00 (2.00, 3.00), and 2.00 (1.00, 3.00), respectively. These scores were significantly lower than the score of 5.00 (4.00, 6.00) recorded before treatment (Z = -5.339, -5.330, -5.278; all P < 0.001). Statistically significant differences were observed between month one and month 3 (Z = -4.914, P < 0.001) and between month three and month 5 (Z = -4.104, P < 0.001). Please refer to **Table 4** for more information on this.

**Table 4:** Pain score related to radioactive ^125^I seed implantation.

Follow-up time	Pain score	Z	P
Preoperative	5.00(4.00,6.00)	–	–
One month	3.00(2.25,4.00)	-5.339	<0.001*
Three months	2.00(2.00,3.00)	-5.330	<0.001*
Five months	2.00(1.00,3.00)	-5.278	<0.001*

*Compared with preoperative.

All 36 patients remained infection-free following ^125^I seed implantation. Two cases of radiation injury were reported: one case of Grade I radiation dermatitis, which gradually improved after treatment with anti-inflammatory drugs, and one case of Grade I radiation pneumonitis, which resolved without specific treatment. Puncture-related complications occurred in two cases. One minor bleeding was controlled by applying local pressure to stop it. In the other case, a small pneumothorax was detected by CT after a few days and resolved spontaneously. Mild myelosuppression was observed in three cases, which was managed with leukocyte-boosting medications and later determined. Five instances of seed displacement were reported, with one involving the left ventricle, one affecting the left upper lung, and three affecting the chest cavity. Radiological toxicity did not occur in these cases, affecting adjacent vital organs. Further details are provided in **Table 5**.

**Table 5:** Complications related to radioactive ^125^I seed implantation (n=36).

Complications	No.	%
Infection	0	0.00
Bleeding	1	2.78
Myelosuppression	3	8.33
Seed migration	5	13.89
Pneumothorax	1	2.78
Radiodermatitis	1	2.78
Radiation pneumonitis	1	2.78

## 4 Discussion

The available studies on the clinical benefit of ^125^I seed implantation for treating RAIR-DTC are limited, and most studies only focus on single types of metastatic cancer, such as lymph nodes or bone metastases (15, 16). This study aims to comprehensively evaluate the clinical improvement of ^125^I seed implantation for RAIR-DTC across a wide range of metastatic sites, including locally recurrent foci in the thyroid surgical bed, metastatic lymph nodes in the neck and mediastinum, lung metastases, bone metastases, and pleural metastases.

This study included a total of 73 RAIR-DTC metastases from various sites. However, 14 lesions that only destroyed bone without soft tissue hyperplasia were excluded because their efficacy could not be evaluated based on tumor size changes. The LER of the remaining 59 metastases was 23.73%, 38.98%, and 52.54% at postoperative months 1, 3, and 5, respectively. The LCR was 98.31%, 96.61%, and 94.92% at postoperative months 1, 3, and 5, respectively. The study’s results align with Chen et al.’s (15) report of a 95% LCR for RAIR-DTC bone metastases in 9 cases. Additionally, the results are superior to Chen et al.’s (16) report of over a 90% shrinkage rate of metastatic lymph nodes in the neck of RAIR-DTC through ultrasound-guided ^125^I seed implantation. The data show that ^125^I seed implantation effectively reduces the tumor size and achieves local tumor control (refer to **Figures 1**, **2**). The physical properties of ^125^I seeds, such as their low energy (27~35 keV) and long half-life (59.5 days), strongly correlate with these results. Due to its low energy and long half-life, the radiation emitted by the ^125^I seeds implanted in the tumor causes minimal damage to surrounding normal tissues while delivering a lethal dose of radiation to the tumor cells. Liyuan Zhang (17) reports that small doses of radiation can stimulate the immune system to suppress tumor growth. Due to the necessity of considering the tolerated dose of surrounding normal tissues, external beam radiotherapy cannot administer a radical dose to tumors. Furthermore, ^125^I particles emit rays continuously over an extended period, impacting the entire cycle of tumor cell division for an extended duration. In contrast, conventional external beam radiotherapy involves multiple split irradiations, which make tumor cells in the interphase of division insensitive to the rays. This is the primary reason why ^125^I seeds can effectively treat cases that have failed conventional external beam radiotherapy, chemotherapy, and ^131^I therapy. Proton and heavy ion radiotherapy can induce double-strand breaks in the chromosomes of tumor cells (18), resulting in a loss of their ability to increase. Due to its unique Bragg peak effect, this effect is achieved with minimal damage to surrounding normal tissues. However, proton and heavy ion therapy are costly and require highly qualified personnel. It is mainly used in clinical research and is still being developed for routine tumor treatment.

The study identified three progressive lesions that may have been caused by the presence of poorly differentiated tumor tissue. These cells have a short doubling time, and the initial dose rate of ^125^I seed is low (approximately 0.07-0.09 Gy/h) (17), which may result in tumor progression before the ^125^I seed has had a chance to take effect. ^125^I seeds can treat tumors by slowly releasing radiation and gradually increasing the cumulative dose in the tumor cells. In this study, most RAIR-DTC tumor cells were relatively inactive and slow-growing. Thus, although the LCR at 1 and 3 months postoperatively were low and most lesions showed SD, the treatment’s effectiveness became more apparent as the cumulative tumor dose increased, consistent with a progressive increase in LCR and LER over time.

Serum Tg is a crucial indicator of postoperative relapse and metastasis in patients with DTC (19). A decrease in serum Tg levels may indicate the effectiveness of DTC treatment if a patient tests negative for TgAb. The study found that the serum Tg levels of the patients decreased after treatment compared to before treatment (both P < 0.001). Additionally, the serum Tg levels of some patients had returned to normal. These findings suggest that ^125^I seeds can effectively inhibit and kill RAIR-DTC tumor cells.

The progression of RAIR-DTC may cause severe pain and a significant reduction in patients’ quality of life, mainly when metastatic lesions occur in the bone and pleura (20). The results of this study indicate that ^125^I seed implantation can effectively relieve and reduce patient pain, resulting in significantly lower pain scores post-treatment compared to pre-treatment.

While ^125^I seed implantation is minimally invasive, it is still considered an invasive treatment. Therefore, it may lead to complications related to puncture, such as bleeding, pain, and pneumothorax. As tumor volume and metastases increase, the number and activity of ^125^I seeds required for implantation also increase, leading to higher radiation exposure and an increased risk of complications. In addition, adverse reactions related to radiation, such as radioactive damage and necrosis, may be experienced (21). Three patients experienced mild bone marrow suppression after ^125^I seed implantation. Patient 1 had 146 particles implanted with an activity of 2.22*10^7^ Bq in an extensive mediastinal lymph node metastasis. In contrast, patients 2 and 3 had 57 and 88 seeds planted with an activity of 2.59*10^7^ Bq in bone metastases with soft tissue formation, respectively. A small number of adverse events were reported in this study, including pneumothorax, bleeding, radiation pneumonitis, dermatitis, and mild myelosuppression. These issues were resolved through observation or symptom management, indicating the relative safety of ^125^I brachytherapy.

## 5 Conclusions

**Figure 3:**
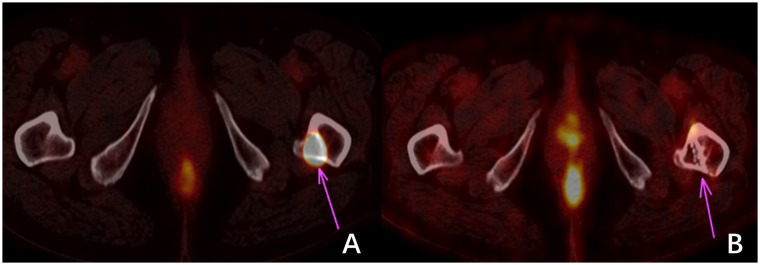
^18^F-FDG imaging of bone metastasis in the left proximal femur of thyroid carcinoma. **(A)** shows a high uptake of 18F-FDG in the metastatic focus before treatment. In contrast, **(B)** shows no significant change in the size of the metastatic focus and bone destruction three months after treatment. However, no 18F-FDG uptake indicates that the tumor cells were inactivated.

^125^I seed brachytherapy has demonstrated reasonable local tumor control, effective pain relief, and minimal side effects in RAIR-DTC. However, this study has several limitations compared to similar research efforts. These limitations include a small sample size, the absence of a control group, and insufficient reliability in data analysis. These limitations hinder a comprehensive depiction of the actual scenario in particle therapy. Furthermore, the follow-up period is relatively short, which limits a thorough evaluation of the medium- and long-term effectiveness of ^125^I brachytherapy (22). This is especially true regarding potential toxic side effects resulting from dose accumulation. The therapy effectiveness evaluation is based solely on changes in tumor dimensions observed through CT scans. However, this approach neglects the consideration of the tumor’s functional metabolism at the treatment site post-implantation (refer to **Figure 3**) or alterations in other tumor sites induced by treatment that may enhance immune responses (23). It is essential to conduct further comprehensive clinical investigations to explore these aspects.

## Data availability statement

The raw data supporting the conclusions of this article will be made available by the authors, without undue reservation.

## Ethics statement

The studies involving humans were approved by Jiangxi Cancer Hospital Ethics Committee. The studies were conducted in accordance with the local legislation and institutional requirements. The participants provided their written informed consent to participate in this study. Written informed consent was obtained from the individual(s) for the publication of any potentially identifiable images or data included in this article.

## Author contributions

QW: Conceptualization, Data curation, Formal analysis, Investigation, Methodology, Software, Writing – original draft. LT: Project administration, Supervision, Validation, Writing – review & editing. XT: Data curation, Investigation, Visualization, Writing – original draft. WW: Funding acquisition, Project administration, Writing – original draft. YS: Investigation, Writing – original draft. ZW: Investigation, Writing – original draft. MK: Investigation, Writing – review & editing. ZC: Funding acquisition, Project administration, Resources, Supervision, Validation, Visualization, Writing – review & editing.
